# TCR-L: an analysis tool for evaluating the association between the T-cell receptor repertoire and clinical phenotypes

**DOI:** 10.1186/s12859-022-04690-2

**Published:** 2022-04-28

**Authors:** Meiling Liu, Juna Goo, Yang Liu, Wei Sun, Michael C. Wu, Li Hsu, Qianchuan He

**Affiliations:** 1 Public Health Sciences Division, Fred Hutchinson Cancer Research Center, Seattle, USA; 2 Department of Mathematics, Boise State University, Boise, USA; 3 Department of Mathematics and Statistics, Wright State University, Dayton, USA

**Keywords:** Association test, CDR3, Clinical phenotypes, T cell receptors, TCR homology, TCR repertoire

## Abstract

**Background:**

T cell receptors (TCRs) play critical roles in adaptive immune responses, and recent advances in genome technology have made it possible to examine the T cell receptor (TCR) repertoire at the individual sequence level. The analysis of the TCR repertoire with respect to clinical phenotypes can yield novel insights into the etiology and progression of immune-mediated diseases. However, methods for association analysis of the TCR repertoire have not been well developed.

**Methods:**

We introduce an analysis tool, TCR-L, for evaluating the association between the TCR repertoire and disease outcomes. Our approach is developed under a mixed effect modeling, where the fixed effect represents features that can be explicitly extracted from TCR sequences while the random effect represents features that are hidden in TCR sequences and are difficult to be extracted. Statistical tests are developed to examine the two types of effects independently, and then the *p* values are combined.

**Results:**

Simulation studies demonstrate that (1) the proposed approach can control the type I error well; and (2) the power of the proposed approach is greater than approaches that consider fixed effect only or random effect only. The analysis of real data from a skin cutaneous melanoma study identifies an association between the TCR repertoire and the short/long-term survival of patients.

**Conclusion:**

The TCR-L can accommodate features that can be extracted as well as features that are hidden in TCR sequences. TCR-L provides a powerful approach for identifying association between TCR repertoire and disease outcomes.

## Introduction

With rapid progress in sequencing technologies, TCR repertoire profiling is emerging as one of the most powerful tools for studying the immune system and its functions [1]. TCRs represent a group of highly polymorphic receptors expressed on the surface of T cells and plays a key role in recognizing antigens presented by the major histocompatibility complex [2]. The high polymorphism of TCRs enables the recognition of a virtually infinite number of antigens and hence is critical to the flexibility of the adaptive immune system. The sequencing of TCRs allows dissection of the TCR repertoire at single-nucleotide resolution and provides unprecedented opportunities to study immune-mediated diseases [3]. For example, the profiling of the TCR repertoire has yielded novel insights into tumor biology and has the potential to answer the current most pressing questions in cancer immunotherapy [4].

TCRs consist of two chains (typically the alpha and beta chains), and the highly diverse repertoire of TCR is the result of the somatic V(D)J recombination mechanism, where V(D)J represents the variable (V), diversity (D), and joining (J) genes. The beta chain is more diverse than the alpha chain due to the involvement of the D gene. The antigen-specificity of TCRs is mainly determined by the complementarity-determining region 3 (CDR3), and the analysis of TCRs is primarily focused on the beta chain’s CDR3 sequences. A snapshot of the TCR data is shown in Table 1. A number of bioinformatic tools have been developed in recent years for processing and analyzing the TCR data, such as the miTCR [5], GLIPH [6], TCR-dist [7], ImmunoMap [8], TCRMatch [9], TRUST4 [10], and AutoCAT [11]. These tools are tremendously important to the analysis of the TCR data and can handle a wide range of tasks, such as the retrieval of TCR sequences from raw data, calculation of V(D)J gene usage within each repertoire, and TCR clustering and epitope prediction. On the other hand, few methods have been developed for conducting the regression analysis for the TCR data, i.e., linking the TCR repertoire with clinical outcomes to interrogate the potential genetic associations. Currently, the most common approach for analyzing the TCR is perhaps to calculate a diversity score for the TCR repertoire, such as the Shannon entropy, and then use this diversity score to conduct the association test with clinical outcomes [12, 13]. However, the diversity score captures only the frequencies of TCR sequences, while a large amount of information, such as the composition of the amino acids and the homology of TCR sequences, is simply ignored. This inefficient use of information can potentially lead to the loss of statistical power and the inability to identify the associations between the TCR repertoire and disease outcomes.

**Table 1:** Data structure of the TCR $\beta$ chain’s CDR3 region

Subj . ID	Nucleotide sequence	Amino acid seq.	Abundance	V segment	$\cdots$
1	TGTGCCAGCAGCTTAGGTCGGGGCAAAGCTTTCTTT	CASSLGRGKAFF	1	TRBV7-9, $\dots$, TRBV11-3	$\cdots$
1	TGTGCCAGCAGTTGGTTAATTGGCTACACCTTC	CASSWLIGYTF	1	TRBV6-4, $\dots$, TRBV6-1	$\cdots$
1	TGTGCCAGCAGCTTAGGACGGGCTGAAGCTTTCTTT	CASSLGRAEAFF	9	TRBV7-9, $\dots$, TRBV11-3	$\cdots$
1	TGTGCCAGCAGCTTGGGTCGATCACCCCTCCACTTT	CASSLGRSPLHF	4	TRBV11-2, $\dots ,$ TRBV11-3	$\cdots$
1	TGTGCCAGCAGCCACGGACGAGCTGAAGCTTTCTTT	CASSHGRAEAFF	2	TRBV4-2	$\cdots$
2	TGTGCCAGCAGGGACAGGCAAGAGACCCAGTACTTC	CASRDRQETQYF	1	TRBV6-4, ..., TRBV6-1	$\cdots$
2	TGTACCTGGAAGGTATTTTTT	CTWKVFF	1	TRBV7-2	$\cdots$
3	TGCAGTGCTAGAGAGCGAGGCGAGCAGTACTTC	CSARERGEQYF	3	TRBV20-1	$\cdots$
4	TGTGCTGTGAGTCAAAACGGTGCCAGACTCATGTTT	CAVSQNGARLMF	1	TRAV8-4, TRAV8-6	$\cdots$
$\vdots$	$\vdots$	$\vdots$	$\vdots$	$\vdots$	$\cdots$

Association analysis of the TCR repertoire is challenging for several reasons. First, there is a large number of TCR sequences in the data and many of the sequences are of low-to-moderate abundance. Hence the data are inherently high-dimensional and sparse. Second, different individuals usually carry a different number of TCR sequences and many of the sequences differ from each other. That is, there are virtually no common features among different subjects. As such, there is no structured $n\times p$ data matrix for analysis (where *n* is the sample size and *p* is the dimension), and traditional statistical methods often do not apply. Third, akin to natural language context, TCR sequences contain many potential features that are difficult to extract, and how to effectively utilize the embedded information is not clear. In addition to the TCR sequences, other biological information, such as biochemical properties of the amino acids, may also facilitate the association analysis with disease outcomes. It is desirable to incorporate external biological information to enhance the power of the TCR repertoire analysis.

In this article, we develop a new analysis tool, TCR-L, for evaluating the association between the TCR repertoire and clinical phenotypes. Our approach is developed under a mixed effect modeling framework, where the fixed effect represents features that can be explicitly extracted from TCR sequences (such as the amino acid composition) while the random effect represents features that are hidden in TCR sequences and are difficult to extract. One advantage of such modeling is that prior biological information can be incorporated into the fixed effect to facilitate the analysis. Another advantage is that the sequence information of TCR repertoire can be utilized. To harness the sequence information, we conduct amino acid sequence alignment for each pair of TCR repertoires and then develop a metric, TCR homology (TCRhom), to characterize the variance matrix of the random effect. Then we adapt the MiST framework [14] to test the fixed effect and the random effect independently. Finally, the *p* values from the two types of effects are combined to yield an overall *p* value for assessing the association between the TCR repertoire and clinical phenotypes. Our approach is motivated by the cancer genome atlas (TCGA)’s study on the immune landscape of cancer [15], where the TCR CDR3 sequences were recovered from RNA-sequencing data of tumor tissues.

We give a detailed description of the proposed approach in the next section. In the Simulation section, we conduct simulations to evaluate the performance of the TCR-L method and compare it to possible alternative approaches. In the Real data analysis section, we apply our proposed approach to a skin cancer dataset from TCGA and show that the TCR-L is able to identify an association between the TCR repertoire and patients’ survival.

## Methods

### Feature extraction

Assume that the total number of subjects is *n*. For $i=1,\dots ,n$, let $R_i$ be the *i*th subject’s TCR repertoire, and $m_i$ be the number of unique amino acid sequences in $R_i$. For $k=1,\dots , m_i,$ let $a_{i,k}$ denote the *k*th unique amino acid sequence, and $w_{i,k}$ denote the abundance of $a_{i,k}.$ The total number of amino acid sequences for $R_i$ is thus $\sum _{k=1}^{m_i}w_{i,k}$. To extract features from the TCR data, it is natural to consider the proportions of amino acids, as these proportions represent basic composition of the TCR repertoire and are potentially related to protein structures. Let *p* denote the total number of amino acids that are presented in the entire TCR dataset. In general, $p=20$ since there are 20 amino acids and they all tend to show up in the dataset. Given $R_i$, let $g_{j}(R_i)$ be the *j*th amino acid’s frequency within the *i*th subject’s TCR repertoire, for $j=1,\dots ,p$. Then, $\frac{g_{j}(R_i)}{\sum _{j=1}^{p}g_{j}(R_i)}$ represents the proportion of the *j*th amino acid for $R_i$. Subsequently, for $i=1,\dots ,n,$ we define the following feature vector for the *i*th subject:

$$\begin{aligned} f(R_i)=\frac{1}{\sum _{j=1}^{p}g_{j}(R_i)}[g_{1}(R_i) \ g_{2}(R_i) \ \cdots \ g_{p}(R_i)]^{\top }. \end{aligned}$$

In other words, $f(R_i)$ is a $p\times 1$ feature vector where each element represents an amino acid’s proportion.

**Fig. 1:**
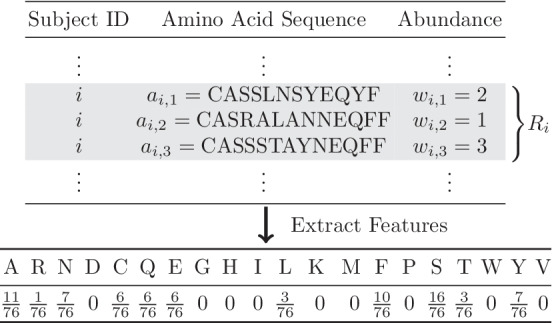
Example of $f(R_i)$ when $p=20$ and $m_i=3$

Figure 1 gives an demonstration on the calculation of $f(R_i)$. Here, the subject *i* contains three unique sequences, $a_{i,1}, a_{i,2}, a_{i,3}.$ The amino acid A (Alanine) occurs once in sequence $a_{i,1}$, thrice in $a_{i,2}$, and twice in $a_{i,3}$. Accounting for the abundance $w_{i,k}$ for $a_{i,k} (k=1,2,3)$, one can calculate $g_{j}(R_i)$ and $f(R_i)$ accordingly. Following the depiction in Fig. 1, we can extract *p* features for *n* subjects and further construct a $n\times p$ feature matrix where each row represents the distribution of *p* amino acids’ proportions corresponding to the subject’s TCR repertoire. Besides the amino acid composition, other potential features, such as the V and J gene usage, can be extracted in a similar manner.

### Amino acid sequence homology

Now suppose that we have one amino acid sequence, $a_{i,k}$, from the *i*th subject’s TCR repertoire, and another sequence, $a_{j,l}$, from the *j*th subject’s TCR repertoire. We wish to align the two sequences and measure the homology between them. The simplest way of aligning sequences and computing the sequence homology is based on the identity matrix which assigns 1 when two amino acids are identical at the aligned position and 0 otherwise. However, this approach does not reflect related, but not identical, amino acids that can be possibly aligned. Alternatively, an amino acid substitution matrix, such as the widely used BLOSUM62 or PAM250 [16], can be used to align a pair of amino acid sequences. The amino acid substitution matrix scores matches and/or mismatches between aligned amino acids more dynamically than the identity matrix.

We perform pairwise alignment via the Needleman-Wunsch algorithm. Default gap penalties were chosen for the alignment algorithm. Let $d(a_{i,k}, a_{j,l})$ be the sum of values on the substitution matrix minus the affine gap penalty for the aligned $a_{i,k}$ and $a_{j,l}$ sequences. Alternatively, the $d(a_{i,k}, a_{j,l})$ can be calculated by other approaches, such as the TCR-dist [7]. Once $d(a_{i,k}, a_{j,l})$ is obtained, the homology between the aligned $a_{i,k}$ and $a_{j,l}$ sequences can be computed as

$$\begin{aligned} s(a_{i,k}, a_{j,l})=\frac{d(a_{i,k}, a_{j,l})}{\sqrt{d(a_{i,k}, a_{i,k})\times d(a_{j,l}, a_{j,l})}}. \end{aligned}$$

The maximum value of $s(a_{i,k}, a_{j,l})$ is 1 when $a_{i,k}$ and $a_{j,l}$ are identical sequences. $s(a_{i,k}, a_{j,l})$ can fall below zero when $d(a_{i,k}, a_{j,l})<0$, when the sum of negative substitution values and gap penalties dominate the sum of positive substitution values. Here, a positive value of the substitution matrix means that the aligned amino acid pair is observed more than expected by chance, whereas a negative one means the opposite [16].

### TCR repertoire homology

Next, we propose a metric, TCR repertoire homology (TCRhom), to measure the homology between two subjects’ TCR repertoires based on $s(a_{i,k}, a_{j,l}), k=1,\ldots , m_i, l=1, \ldots , m_j$. The algorithm of the TCRhom is illustrated in Algorithm 1. Starting with the first amino acid sequence of the *i*th subject’s TCR repertoire, i.e., $a_{i,1}$, pairwise alignments are performed with $a_{j,l}$ $(l=1,\dots ,m_j),$ sequences of the *j*th subject’s TCR repertoire. We compute sequence homology $s(a_{i,1}, a_{j,l})$ for $l=1,\dots ,m_j$ given the substitution matrix and gap penalties. The maximal value of these $s(a_{i,1},a_{j,l}), l=1,\dots ,m_j,$ is identified. Then we multiply the maximal sequence homology by the abundance $w_{i,1}$ that corresponds to $a_{i,1}$. Next, we shift our focus to the second amino acid sequence of the *i*th subject’s TCR repertoire, i.e., $a_{i,2}$. Similarly, pairwise alignments are conducted between $a_{i,2}$ and $a_{j,l}, l=1,\dots ,m_j$, sequences of the *j*th subject’s TCR repertoire. Among the $m_j$ alignments, the maximal sequence homology is identified and then is multiplied by the abundance $w_{i,2}$ accordingly. We repeat this process until every sequence of the *i*th subject’s repertoire has found its maximal homology in the *j*th subject’s repertoire. Once this is completed, we now switch to the *j*th subject’s repertoire $R_j$. Given a sequence $a_{j,l}$ in $R_j$, we find $a_{j,l}$’s maximal homology in $R_i$ and multiply the maximal homology by the abundance $w_{j,l}$. We repeat this procedure until every sequence of $R_j$ has found its maximal homology in $R_i$. A detailed example of the TCRhom calculation is shown in the Additional file 1: Section 1.

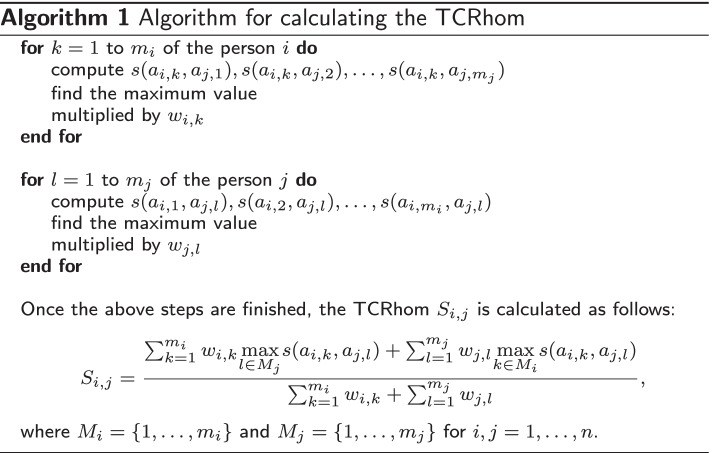



Regardless of which amino acid substitution matrix is used for calculating the homology, the TCRhom preserves two useful properties. First, the TCRhom is symmetric, i.e., ${S}_{i,j}={S}_{j,i}$ for $i,j=1,\dots ,n.$ Second, when $i=j$ (i.e., for the same person), we have $\max _{l \in M_j} s(a_{i,k},a_{j,l}) = \max _{k \in M_i} s(a_{i,k},a_{j,l}) = 1$. Thus, we have ${S}_{i,i}=1$ for $i=1,\dots ,n$, indicating a perfect match between $R_i$ and $R_i$.

To ensure that the TCRhom matrix *S* is positive semi-definite, we use a low-rank approximation via the eigen-decomposition of *S*: ${S}= \sum _{k=1}^{m}\uplambda _k u_k u_k^{\top },$ where $\uplambda _1 \ge \cdots \ge \uplambda _m$ are the non-negative eigenvalues of *S*,  and $u_k,k=1,2,\dots ,m,$ are the corresponding eigenvectors. In what follows, *S* denotes the $n\times n$ symmetric and positive semi-definite TCRhom matrix for *n* subjects.

### Test the association between the TCR repertoire and clinical phenotypes

We adapt the Mixed effects Score Test (MiST) framework [14] to test whether the TCR repertoire is associated with a clinical phenotype. We first introduce some notations. For the *i*th subject, $i=1,\dots ,n$, let $X_i=[1 \ X_{i1} \ X_{i2} \ \cdots \ X_{iq}]^{\top }$ be a $(q+1)\times 1$ vector that consists of an intercept and *q* confounding variables, e.g., patient age, sex, etc. Let $\pi (\cdot )$ be a link function. For continuous traits, $\pi (\cdot )$ is the identity function, and for binary traits, $\pi (x)=e^x/(1+e^x)$. We consider a $n\times 1$ random vector $[h(R_1) \ h(R_2) \ \cdots \ \ h(R_n)]^{\top }$ which is assumed to follow $N(0,\tau ^2 {S})$, where $\tau ^2$ is a scale parameter and *S* can be estimated by the TCRhom matrix. Recall that $R_i$ denotes the *i*th subject’s TCR repertoire, and $f(R_i)$ represents the extracted features of the repertoire, such as the proportions of the 20 amino acids.

Assume a generalized linear mixed model for a clinical phenotype $Y_i$ with its mean

1 $$\begin{aligned} E(Y_i)= \pi \left( \beta ^{\top } X_i+\eta ^{\top }W^{\top }f(R_i)+h(R_i) \right) , \end{aligned}$$

where ${\beta }=[\beta _0 \ \beta _1 \ \beta _2 \ \cdots \ \beta _q]^{\top }$ is a $(q+1)\times 1$ vector of regression coefficients, and $\eta =[\eta _1 \ \eta _2 \ \cdots \ \eta _r]^{\top }$ is a $r\times 1$ vector of regression coefficients. Here, $W=[W_1 \ W_2 \cdots W_r]$ is a $p\times r$ matrix (with $r<p$) and is introduced to accommodate *r* known biological characteristics for $f(R_i)$. For example, hydrophobicity is an important biochemical property of amino acids, and is critical for determining proteins’ structures. To accommodate this property in the proposed model, *W* can be specified as a $p\times 1$ vector whose elements represent the hydrophobicity scores of the 20 amino acids.

We are interested in testing the null hypothesis $H_0: \eta =0$ and $\tau ^2=0,$ that is, neither $f(R_i)$ nor $h(R_i)$ has any influence on the clinical phenotype. Under $H_{0}$, the score for $\eta$ can be derived as

$$\begin{aligned} {U}_{\eta }=\sum _{i=1}^{n}W^{\top }f(R_i)(y_i-{\tilde{\mu }}_{i}), \end{aligned}$$

where ${\tilde{\mu }}_{i}= {\tilde{\beta }}^{\top } X_i$ for a continuous trait and ${\tilde{\mu }}_{i}= \frac{\exp ({\tilde{\beta }}^{\top }X_i)}{1+\exp ({\tilde{\beta }}^{\top }X_i)}$ for a binary trait, and $\tilde{\beta }$ denotes the maximum likelihood estimate (MLE) of $\beta$ under $H_0.$ Let ${\tilde{\Omega }}$ be a $n\times n$ diagonal matrix where the *i*th diagonal element is $\frac{1}{n}\sum _{i=1}^{n}(y_i-{\tilde{\mu }}_i)^{2}$ for the continuous trait and ${\tilde{\mu }}_i(1-{\tilde{\mu }}_i)$ for the binary trait. Let $X=[X_1 \ X_2 \ \cdots \ X_n]^{\top }$ be a $n\times (q+1)$ matrix and ${\tilde{\Gamma }}={\tilde{\Omega }}-{\tilde{\Omega }} X(X^{\top }{\tilde{\Omega }} X)^{-1}X^{\top }{\tilde{\Omega }}$. The asymptotic covariance of $U_{\eta }$ can be estimated by

$$\begin{aligned} {\tilde{\Sigma }}= \sum _{j=1}^{n}\sum _{i=1}^{n}W^{\top }f(R_i){({\tilde{\Gamma }})}_{ij} f^{\top }(R_j)W, \end{aligned}$$

where ${({\tilde{\Gamma }})}_{ij}$ is the (*i*, *j*) element of ${\tilde{\Gamma }}$. Then ${U}_{\eta }^{\top }{{\tilde{\Sigma }}}^{-1}{U}_{\eta }$ asymptotically follows a chi-square distribution with *r* degrees of freedom.

One may also obtain the score for $\tau ^2$ under $H_0$, however, because $f(R_i)$ and $h(R_i)$ are correlated, it is difficult to derive the covariance between the two scores. In line with the MiST approach [14], we consider the null hypothesis $H_{0'}: \tau ^2=0$ without constraining $\eta =0$. Let ${\hat{\gamma }} = (\hat{\beta }^\top , \hat{\eta }^\top )^\top$ denote MLE of $\gamma = ({\beta }^\top , {\eta }^\top )^\top$ under $H_{0'}$. Let $Z_i = (X_i^\top , W^\top f(R_i))^\top$, and let $Z \equiv (Z_1,\ldots , Z_n)^\top$ denote a $n\times d$ matrix, where $d = q+1+r$.

For continuous trait, the score statistic for $\tau ^2$ under $H_{0'}$ can be derived as

$$\begin{aligned} U_{\tau ^2}=(y-{\hat{\mu }})^{\top }S(y-{\hat{\mu }}), \end{aligned}$$

where $y = [y_1, \ldots , y_n]^\top , {\hat{\mu }}=[{\hat{\mu }}_{1} \ {\hat{\mu }}_{2} \ \cdots \ {\hat{\mu }}_{n}]^{\top }$ and ${\hat{\mu }}_{i}= {\hat{\gamma }}^\top Z_i$ for $i = 1, \ldots , n$. Let $P_0 = \text {I}- Z(Z^\top Z)^{-1}Z^\top$. Then it can be shown that $\text {E}(U_{\tau ^2} ) = {{\,\mathrm{tr}\,}}(SP_0)\sigma ^2$. An estimate of $\sigma ^2$ is ${\hat{\sigma }}^2 = \frac{1}{n-d}(y-{\hat{\mu }})^{\top }(y-{\hat{\mu }})$. Furthermore, we can derive that

$$\begin{aligned} \text {Var}\left( U_{\tau ^2} - {{\,\mathrm{tr}\,}}(SP_0){\hat{\sigma }}^2\right) = 2\sigma ^4[{{\,\mathrm{tr}\,}}(SP_0SP_0) - {{\,\mathrm{tr}\,}}(SP_0)^2/(n-d)]. \end{aligned}$$

Then we define the testing statistic to be

$$\begin{aligned} Q_{\tau ^2} = {(U_{\tau ^2} - {{\,\mathrm{tr}\,}}(SP_0)\sigma ^2)}/{\sqrt{2\sigma ^4[{{\,\mathrm{tr}\,}}(SP_0SP_0) - {{\,\mathrm{tr}\,}}(SP_0)^2/(n-d)]}}. \end{aligned}$$

It can be shown that $Q_{\tau ^2}$ asymptotically follows a standard normal distribution under $H_{0'}$.

For binary trait, it is known that the maximization of the log-likelihood is equivalent to iteratively reweighted least squares [17], thus we derive a similar statistic for logistic regression under such a linearization framework. Let ${\hat{\Omega }}$ be a $n\times n$ diagonal matrix where the *i*th diagonal element is ${{\hat{\mu }}_{i}(1-{\hat{\mu }}_{i})}$, where ${\hat{\mu }}_{i} = \frac{\exp ({\hat{\gamma }}^{\top } Z_i)}{\exp ({\hat{\gamma }}^{\top } Z_i) + 1}$. Let $y_{\text {w},i} = {\hat{\gamma }}^{\top }Z_i +\frac{y_i-{\hat{\mu }}_{i}}{{\hat{\mu }}_{i}(1-{\hat{\mu }}_{i})}$ denote the working response for $i = 1, \ldots , n$. Let $Z^{\dagger } = {\hat{\Omega }}^{1/2} Z$ and $y_{\text {w},i}^{\dagger } = {\hat{\Omega }}^{1/2}y_{\text {w},i}$, then the score statistic for $\tau ^2$ under $H_{0'}$ can be derived as

$$\begin{aligned} U_{\tau ^2}^{\dagger } = \left( y^{\dagger }_\text {w}-Z^{\dagger }{\hat{\gamma }}\right) ^{T}S\left( y^{\dagger }_\text {w}-Z^{\dagger }{\hat{\gamma }}\right) , \end{aligned}$$

where $y^{\dagger }_\text {w} = [y^{\dagger }_{\text {w},1},\ldots , y^{\dagger }_{\text {w},n}]^\top$. Let $P_0^{\dagger } = \text {I}- Z^{\dagger }(Z^{\dagger \top }Z^{\dagger })^{-1}Z^{\dagger \top }$. Following the argument in the linear regression, we have $E(U_{\tau ^2}^{\dagger }) = {{\,\mathrm{tr}\,}}(S P_0^{\dagger })\sigma ^{\dagger 2}$. An estimate for $\sigma ^{\dagger 2}$ is ${\hat{\sigma }}^{\dagger 2} = \frac{1}{n-d}(y^{\dagger }_\text {w}-Z^{\dagger }{\hat{\gamma }})^{\top }(y^{\dagger }_\text {w}-Z^{\dagger }{\hat{\gamma }})$. Note that

$$\begin{aligned} \text {Var}\left( U_{\tau ^2}^{\dagger } - {{\,\mathrm{tr}\,}}(SP_0^{\dagger }){\hat{\sigma }}^{\dagger 2}\right) = 2\sigma ^{\dagger 4}\left[ {{\,\mathrm{tr}\,}}\left( SP_0^{\dagger } SP_0^{\dagger }\right) - {{\,\mathrm{tr}\,}}\left( SP_0^{\dagger }\right) ^2/(n-d)\right] . \end{aligned}$$

Let $Q^{\dagger }_{\tau ^2} = {(U^{\dagger }_{\tau ^2} - {{\,\mathrm{tr}\,}}(SP_0^{\dagger })\sigma ^{\dagger 2})}/{\sqrt{2\sigma ^{\dagger 4}[{{\,\mathrm{tr}\,}}(SP_0^{\dagger } SP_0^{\dagger }) - {{\,\mathrm{tr}\,}}(SP_0^{\dagger })^2/(n-d)]}}$. It can be shown that $Q^{\dagger }_{\tau ^2}$ asymptotically follows a standard normal distribution under $H_{0'}$.

One can show that $U_{\tau ^2}$ is independent of $U_{\eta }$, and the proof of the independence is provided in the Additional file 1: Section 2. Thus, the *p* value based on $U_{\eta }$ and the *p* value based on $U_{\tau ^2}$ are independent. Then one can use Fisher’s test statistic to combine the two *p* values. If the combined *p* value is less than the significance level of $\alpha$, then we conclude that the TCR repertoire is associated with the clinical phenotype being studied. We name the proposed approach the TCR-L. Since the *S* matrix can be constructed based on BLOSUM62 or PAM250, this yields two variations for the TCR-L approach, TCRL-B62 and TCRL-P250.

## Simulation

In simulations, we generated TCR sequences of lengths from 10 to 18. We simulated the first four amino acids (i.e., the head segment) of a TCR sequence to be CASS, CASR, CSAR, or CAST, with the probability of 0.7, 0.1, 0.1, or 0.1. We simulated the last two amino acids (i.e., the tail segment) of a TCR sequence to be YF, FF, HF, or TF, with the probability 0.4, 0.4, 0.1, or 0.1. These patterns and probabilities were chosen based on the real data analyzed in the next section. The middle segment was generated by randomly sampling 4–10 amino acids from the 20 amino acids with replacement. Finally, the three segments were concatenated to yield one TCR sequence.

**Fig. 2:**
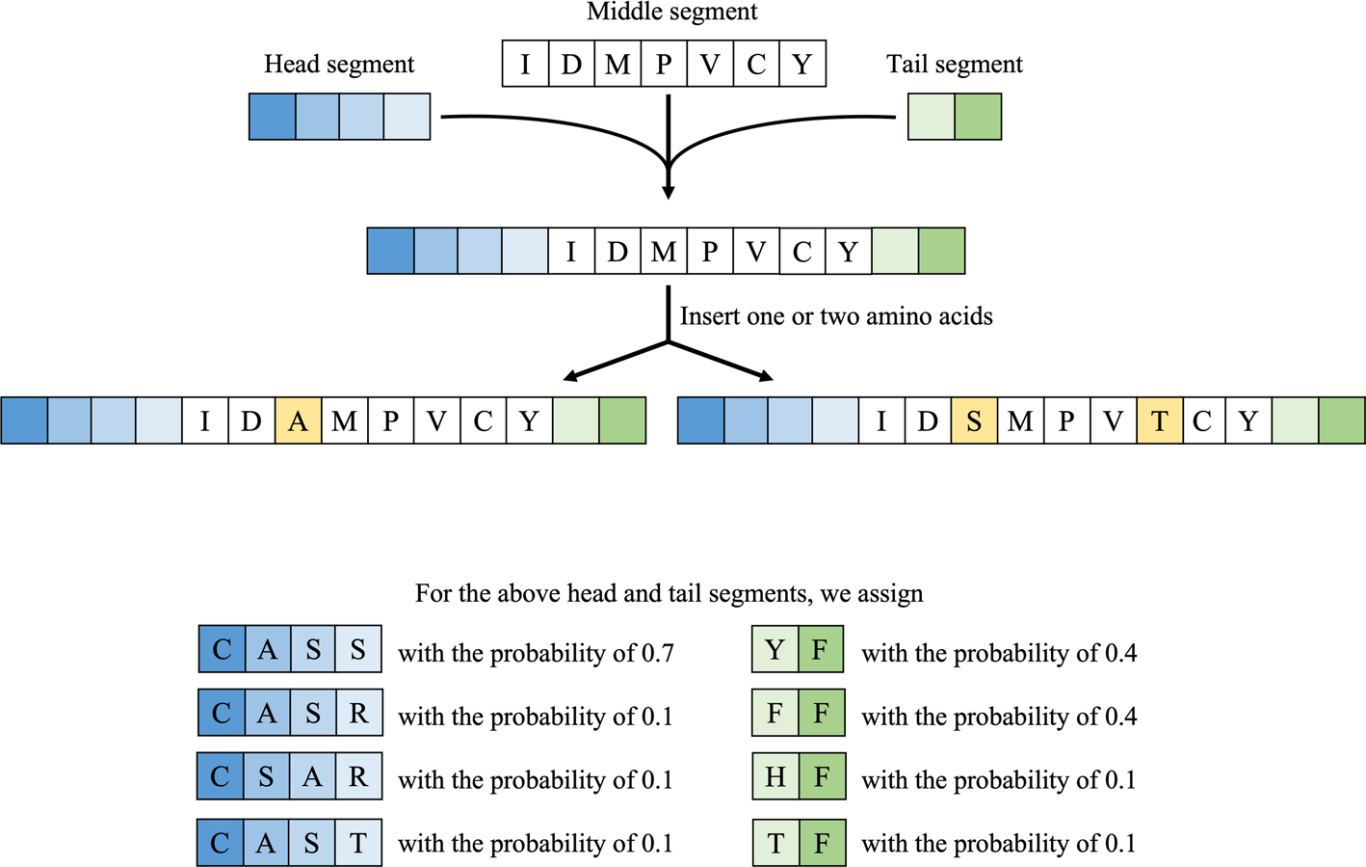
Simulation scheme for generating TCR CDR3 sequences. The yellow boxes represent added amino acids. The blue boxes represent the head segment while the green boxes represent the tail segment of the CDR3 sequence

The diversity of the TCR repertoire is partly due to the P/N nucleotides addition mechanism [18]. Following this mechanism, we randomly introduced 0, 1, or 2 additional amino acids into the middle segment to increase the diversity. An example of the simulation process is given in Fig. 2. To generate the TCR repertoire for the *i*th subject, we randomly drew 2–25 unique TCR sequences for a given subject, and the abundance of each unique sequence was randomly chosen to be 1–5.

Besides the proposed TCR-L approach, we considered two alternative analysis strategies as follows: (I) only the fixed effect (i.e., the effect of the extracted features) was considered; (II) only the random effect (i.e., the effect of the $h(R_i)$) was considered. For (I), we fit the following model

2 $$\begin{aligned} E(y_i)= \pi \left( \beta ^{\top } X_i+\eta ^{\top }W^{\top }f(R_i) \right) , \end{aligned}$$

and then used a score statistic that is similar to ${U}_{\eta }^{\top }{{\tilde{\Sigma }}}^{-1}{U}_{\eta }$ to test the nullity of $\eta$; we call this approach the Ext. features (i.e., extracted features). For (II), we fit the following model

3 $$\begin{aligned} E(y_i)= \pi \left( \beta ^{\top } X_i+h(R_i) \right) , \end{aligned}$$

and then used a statistic similar to $Q_{\tau ^2}$ and $Q_{\tau ^2}^{\dagger }$ to test the effect of $h(R_i)$ for the continuous and the binary traits, accordingly. Depending on whether the *S* matrix was derived from BLOSUM62 or PAM250, this random-effect-based approach was named as the Seq.-B62 or Seq.-P250.

We simulated two confounding variables: $X_{i1}$ followed $\mathrm {Bernoulli}(0.5)$ and $X_{i2}$ followed *N*(0, 1) for $i=1,2,\dots ,n$. We considered the Kyte hydrophobicity of the 20 amino acids for *W*. Then $W^{\top }f(R_i)$ represented the weighted sum of the 20 amino acids’ hydrophobic scores for the *i*th subject. The random effect $h(R_i)$ was generated from $N(0,\tau ^2 S)$, where the variance-covariance matrix *S* was specified based on either the BLOSUM62 or the PAM250. We considered $n=350, 400, 450$. A total of 10,000 and 1,000 replicates were generated for examining type I error and power, respectively. In all simulation studies, the type I error and power of the test were evaluated at the significance level of $\alpha =0.05$.

### Type I error

To evaluate the type I error for each of the methods, we generated a binary trait $y_i, i=1,2,\dots ,n,$ by

$$\begin{aligned} & {\text{logit}}\;P\left( {y_{i} = 1} \right) = \beta _{0} + \beta _{1} X_{{i1}} + \beta _{2} X_{{i2}} , \\ &\;\; {\text{and}}\;{\text{a}}\;{\text{continuous}}\;{\text{trait}}\;y_{i} ,\quad i = 1,2, \ldots ,n,\;{\text{by}} \\ & \;\; y_{i} = \beta _{0} + \beta _{1} X_{{i1}} + \beta _{2} X_{{i2}} + \varepsilon _{i} , \\ \end{aligned}$$

where we set $\beta _0=0.1$, $\beta _1=0.5$, and $\beta _2=-0.4$. The random noise $\varepsilon _i$ was generated from *N*(0, 1). Table 2 shows that the proposed TCRL-P250 and TCRL-B62 were able to control type I error at the nominal level.

**Table 2:** Empirical type I errors ($\times 0.05$) of the TCRL (TCRL-B62 and TCRL-P250) and alternative methods for binary or continuous outcomes

Approach	Binary trait			Continuous trait		
	$n=350$	$n=400$	$n=450$	$n=350$	$n=400$	$n=450$
Ext. features	1.118	1.050	1.008	1.022	1.042	0.956
Seq.-B62	0.980	0.988	0.958	0.938	0.922	0.916
Seq.-P250	0.934	0.944	0.964	0.908	0.890	0.920
TCRL-B62	1.056	1.030	1.050	1.040	1.052	0.980
TCRL-P250	1.014	1.034	1.066	1.028	1.008	0.932

### Power

In all power studies, we fixed $\beta _0=0.1$, $\beta _1=0.5$, and $\beta _2=-0.4$. For the binary trait, we simulated the data such that the sample size per group was no less than 10% of *n* to mitigate the class imbalance problem. In Table 3, we considered the situation that the model contained only fixed effects, that is, we simulated the data under the model (2). For the binary trait, we set $\eta =1.6$, and for the continuous trait, we set $\eta =0.8$. Under this situation, because there was no contribution of the random effect for generating the trait, both the Seq.-P250 and the Seq.-B62 had low power as expected. The power for the TCR-L models was reasonably high, but lower than that of the Ext. features.

**Table 3:** Power comparison of the TCRL and alternative methods for binary or continuous outcomes when only the fixed effect was considered

Approach	Binary trait			Continuous trait		
	$n=350$	$n=400$	$n=450$	$n=350$	$n=400$	$n=450$
Ext.features	0.906	0.920	0.938	0.937	0.950	0.980
Seq.-B62	0.120	0.099	0.114	0.138	0.125	0.159
Seq.-P250	0.112	0.104	0.106	0.126	0.123	0.154
TCRL-B62	0.811	0.842	0.895	0.875	0.911	0.953
TCRL-P250	0.810	0.845	0.892	0.881	0.911	0.951

In Table 4, the binary and continuous traits were simulated under the model (3), where the random effect was generated based on BLOSUM62. We set $\tau ^2=8$ for the binary trait, and $\tau ^2=0.8$ for the continuous trait. As expected, the power for the Seq.-B62 was the highest at each sample size. The power for the Ext. features was the lowest since the fixed effect was not used to generate the trait in this situation. Moreover, we found that the TCRL-P250 maintained good power despite that the true random effect was generated based on the BLOSUM62. This indicates that the proposed approaches are fairly robust to the choice of the amino acid substitution matrix for *S*. Similar results were observed in Table 5 where the random effect was generated based on the PAM250.

**Table 4:** Power comparison of the TCRL and alternative methods for binary or continuous outcomes when only the random effect was considered (*S* was based on BLOSUM62)

Approach	Binary trait			Continuous trait		
	$n=350$	$n=400$	$n=450$	$n=350$	$n=400$	$n=450$
Ext.features	0.094	0.115	0.109	0.092	0.131	0.118
Seq.-B62	0.858	0.919	0.960	0.821	0.891	0.932
Seq.-P250	0.792	0.857	0.917	0.761	0.819	0.884
TCRL-B62	0.822	0.887	0.925	0.780	0.864	0.899
TCRL-P250	0.746	0.818	0.878	0.722	0.783	0.856

**Table 5:** Power comparison of the TCRL and alternative methods for binary or continuous outcomes when only the random effect was considered (*S* was based on PAM250)

Approach	Binary trait			Continuous trait		
	$n=350$	$n=400$	$n=450$	$n=350$	$n=400$	$n=450$
Ext.features	0.123	0.119	0.127	0.099	0.121	0.107
Seq.-B62	0.804	0.881	0.919	0.778	0.861	0.908
Seq.-P250	0.866	0.929	0.962	0.866	0.917	0.949
TCRL-B62	0.765	0.851	0.886	0.744	0.824	0.860
TCRL-P250	0.824	0.907	0.936	0.816	0.890	0.926

Next, we simulated the traits from the model (1), where both the fixed and random effects contributed to the disease outcome. For the binary trait, we set $\eta =1$ and $\tau ^2=6$, and for the continuous trait, we set $\eta =0.5$ and $\tau ^2=0.6$. In Table 6, the random effect was generated based on the BLOSUM62, and in Table 7, the random effect was based on the PAM250. Under this setting, the overall effect was driven by both the fixed effect and the random effect. It can be seen that the power for the TCR-L approaches was among the highest at each sample size in Tables 6 and 7. Similar to the observation in Tables 4 and 5, the TCR-L approaches were quite robust to the choice of the amino acid substitution matrix for *S*. In contrast, the Ext. features, Seq.-B62, and Seq.-P250 had reduced power because they accounted for only one of the two considered effects.

**Table 6:** Power comparison of the TCRL and alternative methods for binary or continuous outcomes when both fixed and random effects were considered (*S* was based on BLOSUM62)

Approach	Binary trait			Continuous trait		
	$n=350$	$n=400$	$n=450$	$n=350$	$n=400$	$n=450$
Ext.features	0.281	0.285	0.324	0.465	0.481	0.528
Seq.-B62	0.809	0.883	0.933	0.751	0.823	0.878
Seq.-P250	0.735	0.801	0.882	0.697	0.758	0.818
TCRL-B62	0.820	0.861	0.922	0.807	0.870	0.916
TCRL-P250	0.743	0.812	0.888	0.777	0.836	0.874

**Table 7:** Power comparison of the TCRL and alternative methods for binary or continuous outcomes when both fixed and random effects were considered (*S* was based on PAM250)

Approach	Binary trait			Continuous trait		
	$n=350$	$n=400$	$n=450$	$n=350$	$n=400$	$n=450$
Ext.features	0.301	0.326	0.321	0.474	0.524	0.541
Seq.-B62	0.761	0.833	0.892	0.711	0.781	0.847
Seq.-P250	0.838	0.906	0.946	0.780	0.845	0.914
TCRL-B62	0.772	0.844	0.892	0.798	0.859	0.895
TCRL-P250	0.832	0.902	0.939	0.843	0.899	0.942

More simulation studies were provided in the Supplementary Material. In Additional file 1: Section 3, we considered two characteristics $W_1$ and $W_2$ for the extracted features. In Additional file 1: Section 4, we conducted simulation studies based on a real dataset of skin cancer. The results of those experiments showed a similar pattern as that in this section.

## Real data analysis

TCR $\beta$-chain’s CDR3 sequences of the skin cutaneous melanoma (SKCM) patients were obtained from TCGA [15]. Patients who had only one unique sequence in their TCR data were excluded. Schadendorf et al. [19] observed that the survival curve of melanoma patients reached a plateau around 3 years of follow-up regardless of prior therapy, ipilimumab dose, or treatment regimen in their clinical trials. Thus, we analyzed a binary trait $y_i$ based on whether the *i*th subject survived at least 3 years ($y_i=0$) or not ($y_i=1$). Those who were censored before 3 years of follow-up were excluded from our analysis. We adjusted for age, gender, tumor purity and ploidy of cancer cells. After removing subjects with missing values, 248 melanoma patients were kept in our analysis, with 67 patients having short-term survival and 181 patients having long-term survival. The total number of unique amino acid sequences across the 248 patients was 6660. The number of unique sequences per patient ranged from 2 to 182, with the median number of 14.5. The length of amino acid sequences ranged from 6 to 15, with the median length of 13.

Prior to fitting our models, we performed the age-adjusted logistic regression to assess the association of the Shannon entropy with the survival status. The Shannon entropy for the *i*th subject was computed as

$$\begin{aligned} \text {Shannon entropy}=-\sum _{k=1}^{m_i}{q}_{i,k}\log {q}_{i,k}, \end{aligned}$$

where ${q}_{i,k}=w_{i,k}/\sum _{k=1}^{m_i}w_{i,k}$ and $w_{i,k}$ was the abundance corresponding to the *k*th unique amino acid sequence. A higher Shannon entropy score translates to a greater diversity for an individual’s TCR repertoire. The results of our logistic regression model show that the odds ratio for age (year) was 1.03 ($95\%$ confidence interval is 1.01–1.05; *p* value: 0.002), suggesting that elder patients were more likely to have short-term survival. As to the Shannon entropy, the association between the Shannon entropy and the survival status after controlling for age has a *p* value of 0.074 (odds ratio: 0.78; $95\%$ confidence interval: 0.60–1.02). This suggests that a higher diversity of the TCR repertoire may be potentially beneficial to longer survival, but the *p* value does not reach the significance level of 0.05.

Next, we applied the proposed approach along with the compared approaches to assess the association between the TCR repertoire and the survival status. We used the proportions of the 20 amino acids as the extracted feature vector $f(R_i)$. To incorporate biochemical properties of amino acids into the analysis, we used the Kyte hydrophobicity score as the *W*. The hydrophobicity score is positive for hydrophobic amino acids and is negative for hydrophilic amino acids. The product of *W* and $f(R_i)$, $W^{\top }f(R_i)$, can be seen as the hydrophobicity score weighted by the proportions of amino acids for *i*th subject’s repertoire. In Table 8, the *p* values for the five considered approaches are listed. It can be seen that the approach based on the extracted features yielded a *p* value of 0.097, indicating that the fixed effect (i.e., the hydrophobicity score) did not have significant association with the survival status. The *p* values for the TCRL-B62 and the TCRL-P250 are 2.75E−03 and 3.52E−03, respectively, both of which are significant at the nominal level of 0.05. The *p* values for the Seq.-B62 and the Seq.-P250 are 2.77E−03 and 3.90E−03, respectively, indicating that the random effect (i.e., the hidden features of the TCR repertoire) made the major contribution to the observed association signal. Overall, our analysis established an association between the TCR repertoire and the survival status. Our findings also suggest that besides features that can be extracted, hidden features should also be considered as they can be important factors for evaluating the association between TCR repertoire and disease outcomes.

**Table 8:** *p* Values for assessing the association between melanoma patients’ TCR repertoires and their survival statuses

Ext. features	Seq.-B62	Seq.-P250	TCRL-B62	TCRL-P250
9.71E−02	2.77E−03	3.90E−03	2.75E−03	3.52E−03

## Discussion

We have proposed an analysis tool for testing the association between the TCR repertoire and clinical phenotypes. Our approach accounts for both the extracted features as well as the features that are difficult to be extracted from the sequences of the TCR repertoire and is able to incorporate prior biological knowledge into the analysis. In our analysis, we have considered the amino acid compositions as the extracted features. Other types of features, such as the amino acid *k*-mers, may be potentially considered as well. The idea of getting *k*-mers is to identify a string of *k* consecutive amino acids, and a similar strategy has been used in text-mining for extracting feature vectors. A major challenge associated with this strategy is that the number of *k*-mers may be relatively large, for example, 400 for the set of 2-mer amino acids. To handle high dimensions, one simple way is to focus on the relatively frequent *k*-mers, though more sophisticated approaches should be further studied. Besides the *k*-mers, new methods have been recently developed to infer cognate targets or antigen specificities of TCRs [20, 21], and these targets/antigens provide potential features for data analysis. With rapid growth of biological knowledge and databases, the prediction of TCR targets is likely to expand dramatically in the coming years. Future development is needed to utilize these predicted targets for feature extraction. As to the sequence homology, the TCRhom was proposed to harness the sequence information embedded in the TCR repertoire, and this approach shares a spirit with related methods in quantitative genetics where the genotype-trait association is modeled through a set of random effects [22, 23]. In quantitative genetics, the variance-covariance matrix of the random effect, *S*, is specified by either the marker-based genetic relationship or the kinship among the studied subjects [24, 25]. In our case, the genetic relationship is characterized by the TCR repertoire, whose composition and abundance vary across different subjects.

We have used the pairwise alignment for aligning amino acid sequences when calculating the TCRhom matrix. Alternatively, multiple sequence alignment (MSA) approaches, such as MAFFT [26], MUSCLE [27], and Clustal Omega [28], can be potentially applied. The MSA methods are usually used to infer conservative regions or evolutionary relationships between the sequences, and generally require higher memory than the pairwise alignment methods. Which methods to use is likely to depend upon specific tasks at hand and the computation environment available to investigators.

As to the limitation of the proposed approach, since the random effect for the TCR repertoire has no explicit features, we can not estimate the coefficients of the features. As such, the size of the association (i.e., regression coefficients) can not be evaluated by using the current model. To obtain the regression coefficients for the association, one would need to extract features from the $R_i$ and evaluate these features under the alternative hypothesis, but how to efficiently extract features from the TCR repertoire remains to be investigated. Another limitation of the proposed approach is with regard to the prediction of the outcome. We note that TCRL is designed to test the genetic associations for TCR repertoire and is not yet able to be used for predicting the outcome. To do prediction, one usually needs to have a set of features as well as the estimated sizes for these features. The TCRL does not have these needed components and we hope that future research on feature extraction would help to address this important issue.

There remain challenges to analyze the TCR repertoire. For example, how to combine the $\alpha$ and $\beta$ chains of the TCR to conduct a more comprehensive analysis is largely unknown. Moreover, many neoantigens in cancers are caused by somatic mutations, and how to harness such information in the TCR analysis is still unclear. One potential strategy is to infer the neoantigens using the somatic mutations of the patients, and then investigate if there are specific TCRs that target these antigens. Thus, somatic mutations and TCR repertoire are inherently related to each other, and the joint analysis of them can potentially provide important information for immunotherapy. Finally, while our simulation studies and real data analysis are focused on the TCR analysis, the proposed approach can be also applied to the analysis of B cell receptor repertoire, which has been shown to play important roles in immune-mediated diseases [29]. Overall, our approach provides a new tool to examine the relationship between the immune repertoire and disease outcomes.

## Conclusions

To conclude, we have developed an approach, TCR-L, that is able to utilize both extracted features and hidden features to test the association between the TCR repertoire and clinical outcomes. Through simulations, we showed that our proposed approach controls the type I error well and is more powerful than possible alternative approaches. In real data analysis, our approach successfully identified an association between the TCR repertoire and the survival of melanoma patients, demonstrating the power of the proposed approach. Overall, the TCR-L provides a timely tool for examining the association between the TCR repertoire and clinical outcomes, which has emerged as an important topic in clinical investigations.

## Supplementary Information

**Additional file 1: **Supplementary material, including a detailed example of the TCRhom calculation, the proof of independence of score statistics and more simulation studies.

## Data Availability

The tcrl R package is available at https://github.com/rksyouyou/TCRL-pkg. Simulation code is available on https://github.com/rksyouyou/TCRL-simulation. Data are available from the GDC Data Portal https://portal.gdc.cancer.gov/ upon approval.

## Abbreviations

TCRs: T cell receptors
TCR: T cell receptor
TCRhom: TCR homology
TCGA: the cancer genome atlas
MLE: maximum likelihood estimate
SKCM: skin cutaneous melanoma
MSA: multiple sequence alignment
