# Supplementary material for: TCR-L: an analysis tool for evaluating the association between the T-cell receptor repertoire and clinical phenotypes
Source: BMC Bioinformatics. 2022 Apr 28;23:152. doi: 10.1186/s12859-022-04690-2 (PMC9052542; doi:10.1186/s12859-022-04690-2)
Supplement: Supplementary file 1 — Additional file 1: Supplementary material, including a detailed example of the TCRhom calculation, the proof of independence of score statistics and more simulation studies. [file 12859_2022_4690_MOESM1_ESM.pdf]

Supplementary Material for
"TCR-L: an analysis tool for evaluating the association between the T-cell receptor repertoire and clinical phenotypes"

Meiling Liu[1], Juna Goo[2], Yang Liu[3], Wei Sun[1], Michael C Wu[1], Li Hsu[1] and Qianchuan He[1]

[1] Public Health Sciences Division, Fred Hutchinson Cancer Research Center, Seattle, U.S.A.

[2] Department of Mathematics, Boise State University, Boise, U.S.A.

[3] Department of Mathematics and Statistics, Wright State University, Dayton, U.S.A.

1 Demonstration of the TCRhom computation

Supplementary Table S1: Basic example of the TCR data

Subject ID	Amino Acid Sequence	Abundance
1	CASSYSVGTDTQYF	1
1	CASSDGGGNEQFF	4
1	CASSLIRNGYT	2
2	CASSELLTGYTF	3
2	CASSYGFRWGGEQYF	1

Table S1 shows a simple data structure which contains two subjects' repertoires R_1 and R_2. The first subject's repertoire R_1 has three unique amino acid sequences $a_{1,1}$ = CASSYSVGTDTQYF, $a_{1,2}$ = CASSDGGGNEQFF, and $a_{1,3}$ = CASSLIRNGYT. The corresponding abundances are $w_{1,1} = 1, w_{1,2} = 4$, and $w_{1,3} = 2$. The second subject's repertoire R_2 has two unique amino acid sequences $a_{2,1}$ = CASSELLTGYTF and $a_{2,2}$ = CASSYGFRWGGEQYF with the abundances $w_{2,1} = 3$ and $w_{2,2} = 1$, respectively. For the computation of $s(a_{1,k}, a_{2,l}), k = 1, 2, 3, l = 1, 2$, we use the BLOSUM62 substitution matrix with the default affine gap penalty for pairwise alignments via the Needleman-Wunsch algorithm. The affine gap penalty is referred to as gap opening + gap extension × length of gaps, where the gap opening is the cost of creating a gap ($-$) and the gap extension is the cost of extending the gap.

Then the TCRhom $S_{1,2}$ is computed as

$$S_{1,2} = \frac{\sum_{k=1}^{3} w_{1,k} \max_{l\in\{1,2\}} s(a_{1,k}, a_{2,l}) + \sum_{l=1}^{2} w_{2,l} \max_{k\in\{1,2,3\}} s(a_{1,k}, a_{2,l})}{\sum_{k=1}^{3} w_{1,k} + \sum_{l=1}^{2} w_{2,l}} = 0.3544903.$$

Likewise, it is easy to show that $S_{2,1} = 0.3544903$. Thus, we say that the homology between two subjects' TCR repertoires is 0.3544903.

2 Independence between U_η and U_{τ^2}

Let

$$X = \begin{bmatrix} 1 & X_{11} & X_{12} & \cdots & X_{1q} \\ 1 & X_{21} & X_{22} & \cdots & X_{2q} \\ \vdots & \vdots & \vdots & \vdots & \\ 1 & X_{n1} & X_{n2} & \cdots & X_{nq} \end{bmatrix} \quad : n \times (q+1) \text{ matrix}$$

and

$$Z = \begin{bmatrix} 1 & X_{11} & X_{12} & \cdots & X_{1q} & W_1^\top f(R_1) & W_2^\top f(R_1) & \cdots & W_r^\top f(R_1) \\ 1 & X_{21} & X_{22} & \cdots & X_{2q} & W_1^\top f(R_2) & W_2^\top f(R_2) & \cdots & W_r^\top f(R_2) \\ \vdots & \vdots & \vdots & \vdots & \vdots & \vdots & \vdots & \vdots & \vdots \\ 1 & X_{n1} & X_{n2} & \cdots & X_{nq} & W_1^\top f(R_n) & W_2^\top f(R_n) & \cdots & W_r^\top f(R_n) \end{bmatrix} \quad : n \times (q+1+r) \text{ matrix,}$$

where $f(R_i)$ is a $p \times 1$ vector of the extracted features of the ith subject's TCR repertoire R_i and $W = [W_1 \ W_2 \cdots W_r]$ is a $p \times r$ matrix whose $W_l, l = 1, 2, \ldots, r$, is a $p \times 1$ vector of amino acid characteristic corresponding to the p amino acids of the feature vector. We adapt the proof in the MiST (Sun et al., 2013) framework to prove the independence between U_{τ^2} and U_η.

Let $A = I - \Omega X(X^\top \Omega X)^{-1} X^\top$ and $B = I - \Omega Z(Z^\top \Omega Z)^{-1} Z^\top$, where Ω is a $n \times n$ diagonal variance matrix of y and I is a $n \times n$ identity matrix. By Taylor's expansion, we can approximate

$$\begin{bmatrix} y_1 - \tilde{\mu}_1 \\ y_2 - \tilde{\mu}_2 \\ \vdots \\ y_n - \tilde{\mu}_n \end{bmatrix} \approx \begin{bmatrix} A_{11} & \cdots & A_{1n} \\ A_{21} & \cdots & A_{2n} \\ \vdots & \vdots & \vdots \\ A_{n1} & \cdots & A_{nn} \end{bmatrix} \begin{bmatrix} y_1 - \mu_1 \\ y_2 - \mu_2 \\ \vdots \\ y_n - \mu_n \end{bmatrix} \quad \text{and} \quad \begin{bmatrix} y_1 - \hat{\mu}_1 \\ y_2 - \hat{\mu}_2 \\ \vdots \\ y_n - \hat{\mu}_n \end{bmatrix} \approx \begin{bmatrix} B_{11} & \cdots & B_{1n} \\ B_{21} & \cdots & B_{2n} \\ \vdots & \vdots & \vdots \\ B_{n1} & \cdots & B_{nn} \end{bmatrix} \begin{bmatrix} y_1 - \mu_1 \\ y_2 - \mu_2 \\ \vdots \\ y_n - \mu_n \end{bmatrix}.$$

We consider the following eigen-decomposition of S: $S = \sum_{k=1}^m \lambda_k \, u_k \, u_k^\top$, where $\lambda_1 \geq \cdots \geq \lambda_m$ are the m non-negative eigenvalues of S and $u_k = [u_{k1} \ u_{k2} \ \cdots \ u_{kn}]^\top$ is the $n \times 1$ corresponding eigenvector for $k = 1, 2, \ldots, m$. Let $\xi_i = [\sqrt{\lambda_1} u_{1i} \ \sqrt{\lambda_2} u_{2i} \ \cdots \ \sqrt{\lambda_m} u_{mi}]^\top$ for $i = 1, 2, \ldots, n$. We can write

$$S = \begin{bmatrix} \xi_1^\top \\ \xi_2^\top \\ \vdots \\ \xi_n^\top \end{bmatrix} \begin{bmatrix} \xi_1 \ \xi_2 \ \cdots \ \xi_n \end{bmatrix},$$

and hence, $U_{\tau^2} = (y - \widehat{\mu})^\top S(y - \widehat{\mu}) = \sum_{i=1}^n \sum_{j=1}^n (y_j - \widehat{\mu}_j)\xi_j^\top \xi_i(y_i - \widehat{\mu}_i) = D_{\tau^2}^\top D_{\tau^2}$, where $D_{\tau^2} = \sum_{i=1}^n \xi_i(y_i - \hat{\mu}_i)$. Then we have

$$U_\eta = \sum_{i=1}^n W^\top f(R_i)(y_i - \tilde{\mu}_i) \approx \sum_{i=1}^n W^\top f(R_i) \sum_{j=1}^n A_{ij}(y_j - \mu_j), \quad : \text{a } r \times 1 \text{ vector}$$
$$D_{\tau^2} = \sum_{i=1}^n \xi_i(y_i - \hat{\mu}_i) \approx \sum_{i=1}^n \xi_i \sum_{j=1}^n B_{ij}(y_j - \mu_j) \quad : \text{a } m \times 1 \text{ vector.}$$

Hence, we get

$$\begin{aligned}
\mathrm{Cov}(U_\eta, D_{\tau^2}) &\approx \mathrm{Cov}(\sum_{i=1}^n \sum_{j=1}^n W^\top f(R_i) A_{ij}(y_j - \mu_j), \sum_{i=1}^n \sum_{j=1}^n \xi_i B_{ij}(y_j - \mu_j)) \\
&= \sum_{i=1}^n \sum_{j=1}^n W^\top f(R_i) A_{ij} \Omega_{jj} B_{ij} \xi_i^\top, \\
&= \sum_{i=1}^n \sum_{j=1}^n W^\top f(R_i) A_{ij} B_{ij} \Omega_{jj} \xi_i^\top \\
&= \sum_{i=1}^n \sum_{j=1}^n W^\top f(R_i)[I_{ij} - (\Omega X(X^\top \Omega X)^{-1} X^\top)_{ij}] B_{ij} \Omega_{jj} \xi_i^\top.
\end{aligned}$$

Note that $Z^\top B\Omega = Z^\top\Omega - Z^\top\Omega Z(Z^\top\Omega Z)^{-1}Z^\top\Omega = Z^\top\Omega - Z^\top\Omega = 0_{(q+1+r)\times n}$. That is, we have

- $\sum_{i=1}^n B_{ij}\Omega_{jj} = 0$ for $j = 1, 2, \ldots, n$.
- $\sum_{i=1}^n X_{ik} B_{ij}\Omega_{jj} = 0$ for $k = 1, 2, \ldots, q$ and $j = 1, 2, \ldots, n$.
- $\sum_{i=1}^n W_l^\top f(R_i) B_{ij}\Omega_{jj} = 0$ for $l = 1, 2, \ldots, r$ and $j = 1, 2, \ldots, n$.

Thus, $\mathrm{Cov}(U_\eta, D_{\tau^2})$ is a $r \times m$ matrix of zeros. Since U_{τ^2} is solely based on D_{τ^2}, hence U_{τ^2} is independent of U_η.

3 Additional simulation studies

For each of the sample sizes ($n = 350, 400, 450$), $1,000$ simulations were performed. Two confounding variables were generated: for $i = 1, 2, \ldots, n$, X_{i1} followed a Bernoulli distribution with the probability of 0.5 and X_{i2} followed a standard normal distribution. We simulated two characteristics W_1 and W_2 as follows: W_1 was a 20×1 vector whose the elements were uniformly distributed from -3 through 3 and W_2 was a 20×1 vector whose the elements were normally distributed with a mean of 0 and a variance of 4. We used a 20×1 vector for $f(R_i), i = 1, 2, \ldots, n,$. We considered the fixed effects $W_1^\top f(R_i)$ and $W_2^\top f(R_i)$ for $i = 1, 2, \ldots, n$. The random effect $[h(R_1)\ h(R_2)\ \cdots\ h(R_n)]^\top$ followed a multivariate normal distribution with a mean vector of 0s and a variance-covariance matrix $\tau^2 S$, where S was specified based on either BLOSUM62 or PAM250. For $i = 1, 2, \ldots, n$, ε_i followed a standard normal distribution.

Then we considered the following TCR-L models: for the binary trait $y_i, i = 1, 2, \ldots, n$,

$$\text{logit} P(y_i = 1) = \beta_0 + \beta_1 X_{i1} + \beta_2 X_{i2} + \eta_1 W_1^\top f(R_i) + \eta_2 W_2^\top f(R_i) + h(R_i),$$

and for the continuous trait $y_i, i = 1, 2, \ldots, n$,

$$y_i = \beta_0 + \beta_1 X_{i1} + \beta_2 X_{i2} + \eta_1 W_1^\top f(R_i) + \eta_2 W_2^\top f(R_i) + h(R_i) + \varepsilon_i.$$

The power for the TCR-L models was evaluated where we set

- $\beta_0 = 0.1$, $\beta_1 = 0.5$, $\beta_2 = -0.4$, $\eta_1 = 3$, $\eta_2 = 1.5$, and $\tau^2 = 4$ for the binary trait
- $\beta_0 = 0.1$, $\beta_1 = 0.5$, $\beta_2 = -0.4$, $\eta_1 = 1.5$, $\eta_2 = 0.5$, and $\tau^2 = 0.5$ for the continuous trait.

Here, both the fixed and random effects contributed to the trait. The variance-covariance matrix of the random effect, S, was specified based on the PAM250 in Table S2, and hence, the power for the TCRL-P250 was the highest at each sample size. A comparable power was observed in the TCRL-B62 in Table S2, indicating that the TCR-L approaches are fairly robust to the choice of substitution matrix for S. This finding is consistent in Table S3 when S was specified based on BLOSUM62. Table S3 shows that the TCRL-B62 has the highest power and the TCRL-P250 has a comparable power.

Supplementary Table S2: Power comparison of the TCRL and alternative methods for binary or continuous outcomes when both the fixed and random effects were considered (S was based on PAM250)

	Binary Trait			Continuous Trait		
Approach	$n = 350$	$n = 400$	$n = 450$	$n = 350$	$n = 400$	$n = 450$
Ext. features	0.527	0.613	0.633	0.735	0.790	0.839
Seq.-B62	0.695	0.768	0.809	0.661	0.711	0.798
Seq.-P250	0.762	0.836	0.884	0.712	0.783	0.864
TCRL-B62	0.807	0.879	0.907	0.863	0.917	0.955
TCRL-P250	0.855	0.918	0.947	0.888	0.938	0.970

Supplementary Table S3: Power comparison of the TCRL and alternative methods for binary or continuous outcomes when both the fixed and random effects were considered (S was based on BLOSUM62)

	Binary Trait			Continuous Trait		
Approach	$n = 350$	$n = 400$	$n = 450$	$n = 350$	$n = 400$	$n = 450$
Ext.features	0.520	0.598	0.611	0.749	0.800	0.821
Seq.-B62	0.737	0.783	0.875	0.695	0.758	0.828
Seq.-P250	0.674	0.726	0.810	0.639	0.698	0.770
TCRL-B62	0.841	0.887	0.940	0.880	0.946	0.961
TCRL-P250	0.788	0.853	0.904	0.849	0.911	0.939

Alternatively, we also evaluated the power when only the fixed effect was considered. In this case, we set

- $\beta_0 = 0.1$, $\beta_1 = 0.5$, $\beta_2 = -0.4$, $\eta_1 = 3$, $\eta_2 = 2$, and $\tau^2 = 0$ for the binary trait
- $\beta_0 = 0.1$, $\beta_1 = 0.5$, $\beta_2 = -0.4$, $\eta_1 = 1.5$, $\eta_2 = 1$, and $\tau^2 = 0$ for the continuous trait.

Supplementary Table S4: Power comparison of the TCRL and alternative methods for binary or continuous outcomes when only the fixed effect was considered

	Binary Trait			Continuous Trait		
Approach	$n = 350$	$n = 400$	$n = 450$	$n = 350$	$n = 400$	$n = 450$
Ext.features	0.923	0.956	0.974	0.943	0.977	0.984
Seq.-B62	0.081	0.069	0.075	0.087	0.086	0.115
Seq.-P250	0.085	0.077	0.100	0.098	0.108	0.123
TCRL-B62	0.850	0.910	0.942	0.898	0.944	0.959
TCRL-P250	0.851	0.909	0.945	0.890	0.935	0.962

Given that only the fixed effect was associated with the trait, the power for the Ext. features was the highest. The power for the TCR-L approaches was adequate since the TCR-L approach included the fixed effect.

4 More simulations based on real data

In this section, we conducted simulation studies based on the skin cutaneous melanoma (SKCM) dataset in TCGA. The data have been described in the Real Data Analysis section in the main text. We generated two confounding variables X_1 and X_2 to emulate gender and age in the real data. In detail, the X_1 was generated from a Bernoulli distribution with the probability parameter being 0.66, where 0.66 is the proportion of male subjects in the SKCM data. To mimic the distribution of the age in the real data, we generated X_2 from a normal distribution with mean 56 and standard deviation 16. The age observed from the SKCM data was between 15 and 86; thus, if the simulated age was out of this range, we re-generated X_2 from the normal distribution until X_2 falls into the range. For the parameter setup, we observed from the real data analysis that the regression coefficients for intercept, gender and age were $\hat{\beta}_0 = -2.9, \hat{\beta}_1 = 0.23$, and $\hat{\beta}_2 = 0.034$, respectively, thus we used these values for the parameters in our simulations.

To mimic the S matrix in the real data, we directly sampled the TCR repertoire from the subjects in the SKCM data. In other words, the TCR repertoire R_i was not generated through the simulation scheme described in the main text, but from the real TCR repertoire of the SKCM patients. After removing subjects with a single amino acid sequence, we had 349 subjects in the SKCM dataset. Then, we sampled R_i for $i = 1, 2, \ldots, n$ from the TCR repertoires of the 349 subjects without replacement for each simulation replicate. Next, the weighted hydrophobic score for the ith subject, $W^T f(R_i)$, and the TCRhom matrix, S, were computed accordingly. Once the S was computed, the random effects $[h(R_1), \ldots, h(R_n)]^T$ were generated from $N(0, \tau^2 S)$.

We generated a binary trait $y_i, i = 1, 2, \ldots, n$, by

$$\text{logit}P(y_i = 1) = \beta_0 + \beta_1 X_{i1} + \beta_2 X_{i2} + \eta W^T f(R_i) + h(R_i),$$

and a continuous trait $y_i, i = 1, 2, \ldots, n$, by

$$y_i = \beta_0 + \beta_1 X_{i1} + \beta_2 X_{i2} + \eta W^T f(R_i) + h(R_i) + \epsilon_i,$$

where ϵ_i was generated from $N(0, 1)$.

We considered three scenarios for power comparison: the model contained only fixed effect, the model contained only random effect, and the model contained both fixed and random effects. These three scenarios were denoted as SI, SII, and SIII, respectively. The parameter settings for SI, SII, and SIII are given in Table S5. For SII and SIII, depending on whether the S matrix was derived from BLOSUM62 or PAM250, the data simulation schemes were named as SII-B62, SIII-B62, or SII-P250, SIII-P250.

Supplementary Table S5: Parameter settings for SI, SII, and SIII for the binary or continuous trait

Scenario	Binary trait	Continuous trait
SI	$\eta = 2.5, \tau = 0$	$\eta = 1, \tau = 0$
SII	$\eta = 0, \tau = 4$	$\eta = 0, \tau = 1$
SIII	$\eta = 2, \tau = 2$	$\eta = 0.8, \tau = 0.8$

We considered $n = 250$ and 300, and simulated the data such that the sample size per group was no less than 10% of n. We replicated 1,000 times and evaluated the power at the significance level of $\alpha = 0.05$. The results of the power comparison were summarized in Tables S6 - S9. These results showed that the TCRL had a robust performance in SI and SII and was the most competitive approach in SIII. The observed pattern of power was similar to that of the main text.

Supplementary Table S6: Power comparison of the TCRL and alternative methods for binary outcomes for situations SI, SII, and SIII ($n = 250$)

	SI	SII-B62	SII-P250	SIII-B62	SIII-P250
Ext.features	0.953	0.157	0.143	0.641	0.625
Seq.-B62	0.173	0.896	0.892	0.706	0.689
Seq.-P250	0.174	0.872	0.916	0.677	0.718
TCRL-B62	0.914	0.871	0.867	0.842	0.827
TCRL-P250	0.911	0.840	0.889	0.819	0.837

Supplementary Table S7: Power comparison of the TCRL and alternative methods for continuous outcomes for situations SI, SII, and SIII ($n = 250$)

	SI	SII-B62	SII-P250	SIII-B62	SIII-P250
Ext.features	0.960	0.152	0.126	0.679	0.694
Seq.-B62	0.156	0.846	0.833	0.756	0.750
Seq.-P250	0.155	0.816	0.863	0.736	0.775
TCRL-B62	0.902	0.814	0.796	0.869	0.884
TCRL-P250	0.901	0.797	0.828	0.859	0.892

Supplementary Table S8: Power comparison of the TCRL and alternative methods for binary outcomes for situations SI, SII, and SIII ($n = 300$)

	SI	SII-B62	SII-P250	SIII-B62	SIII-P250
Ext.features	0.981	0.164	0.159	0.685	0.686
Seq.-B62	0.223	0.936	0.929	0.757	0.730
Seq.-P250	0.213	0.910	0.951	0.728	0.765
TCRL-B62	0.956	0.920	0.916	0.879	0.872
TCRL-P250	0.953	0.891	0.938	0.862	0.887

Supplementary Table S9: Power comparison of the TCRL and alternative methods for continuous outcomes for situations SI, SII, and SIII ($n = 300$)

	SI	SII-B62	SII-P250	SIII-B62	SIII-P250
Ext.features	0.984	0.135	0.134	0.734	0.738
Seq.-B62	0.167	0.923	0.884	0.856	0.828
Seq.-P250	0.152	0.904	0.912	0.835	0.857
TCRL-B62	0.954	0.904	0.854	0.930	0.932
TCRL-P250	0.951	0.873	0.878	0.922	0.944
